# Increased mortality in dementia patients using inhaled anticholinergics: A nationwide register study from the Swedish registry on dementia/cognitive disorders, SveDem

**DOI:** 10.1177/13872877251406309

**Published:** 2025-12-16

**Authors:** Suzan Al-Mayahi, Marine L Andersson, Minjia Mo, Sara Garcia-Ptacek, Hong Xu, Eva Wikström, Maria Eriksdotter

**Affiliations:** 1 Division of Clinical Pharmacology, Department of Laboratory Medicine, Karolinska Institutet and Clinical Pharmacology, Medical Diagnostics Karolinska, Karolinska University Hospital, Stockholm, Sweden; 2 Division of Clinical Geriatrics, Department of Neurobiology, Care Sciences and Society, Karolinska Institute, Stockholm, Sweden; 3 Theme Inflammation and Aging, Medical unit Aging, Karolinska University Hospital, Huddinge, Sweden

**Keywords:** Alzheimer's disease, asthma, cholinesterase inhibitor, chronic obstructive pulmonary disease, cognitive decline, dementia, inhaled anticholinergics

## Abstract

**Background:**

Patients with chronic obstructive pulmonary disease (COPD) face increased risks of cognitive impairment and mortality compared with the general population. Inhaled anticholinergics (LAMA/SAMA) are central in COPD treatment. The link between COPD and dementia is well studied, while effects of COPD medications on survival in dementia patients, have received limited attention.

**Objective:**

Describe dementia patients using LAMA/SAMA in the Swedish Dementia Registry (SveDem) and compare survival between users (exposed) and non-users (unexposed).

**Methods:**

This register-based study used data from SveDem and the Swedish Prescribed Drug Register to identify dementia patients using inhaled anticholinergics. All patients diagnosed with dementia between 2008-01-01 and 2017-12-31 were included. Exposed patients had at least one LAMA/SAMA dispensation per year in the two years prior the index date or more than one in the year before. Standardized-mortality-rates (SMR) were calculated, and survival analysed using Kaplan-Meier and Cox regression.

**Results:**

A total of 74,018 dementia patients were included, of whom 3.5% had used inhaled anticholinergics. Alzheimer's disease was the most common dementia type. SMR was higher in exposed patients across all age groups: 8.21 versus 4.08 (ages 61–75) and 2.94 versus 1.84 (ages 75–90). Exposed had a higher risk of death (crude HR 1.73, 95% CI: 1.62–1.86) compared to unexposed.

**Conclusions:**

In this register-based study we observed an association between inhaled anticholinergic use and reduced survival in dementia patients. This association is thought to be mainly driven by the underlying disease, COPD. Further studies are needed to clarify effects of inhaled anticholinergics on survival.

## Introduction

Chronic obstructive pulmonary disease (COPD) is characterized by respiratory symptoms and irreversible obstructivity which increase over time.^1^ COPD is the third cause of death worldwide^2^ and is reported to occur in 8–10% of the population in Sweden.^3^ Severity of COPD and comorbidities are strongly associated with earlier death in these patients.^4^ Hypoxemia in COPD is related to cognitive impairment and comorbidity in COPD and dementia is common (32%).^5,6^ The relationship between COPD and dementia may be further explained by increased systemic inflammation and cytokines that contribute to neuron damage and neurodegeneration.^5^

According to the Global Initiative for Chronic Obstructive Lung Disease (GOLD),^1^ pharmacotherapy of COPD is currently based on inhaled bronchodilators such as long-acting muscarinic receptor-antagonists (LAMA) and short-acting muscarinic receptor-antagonists (SAMA), together called inhaled anticholinergics. In Sweden, LAMA has only been recommended for maintenance treatment of asthma since 2015 and the recommendations have hitherto been limited to use in more severe asthma.^3,7^ In Sweden, SAMA is often administered in acute asthma in emergency rooms, but not generally recommended for daily treatment.^7^ In a Swedish context, use of inhaled anticholinergics may therefore be used as a proxy for COPD diagnosis.

Dementia is a clinical syndrome caused by neurodegenerative processes, partly attributed to deficits in the cholinergic neurotransmitter system,^8^ that contribute to irreversible cognitive decline.^9^ Increased age in the population has contributed to an increased number of people with dementia. The world-wide prevalence is expected to triple by 2050.^10,11^ There are several dementia disorders of which Alzheimer's disease is the most common. Many dementia patients have variable symptoms, mixed dementia is therefore often diagnosed.^12^

Dementia is the 7:th cause of death worldwide.^13^ The mortality rate differs between different dementia disorders^14^ and dementia patients also survive shorter than the general population does.^14,15^ COPD patients survive approximately 8.3 years shorter than the general population does.^4^

Patients with cognitive impairment often face difficulties with dosing and proper inhalation technique for inhaled drugs against obstructive lung disease, which can result in under- or overmedication.^16^ High concentrations of LAMA may increase pulmonary absorption and thereby increase the risk of systemic side-effects. In addition, elderly patients are at risk of increased drug exposure due to drug-drug interactions and physiologically altered drug metabolism.^17^ Thus, drug exposure in elderly dementia patients may differ significantly from that in younger patients.

It's well established that anticholinergics reaching the CNS may elevate the risk of cognitive impairment and mortality in the elderly in general and specifically in dementia patients.^18,19^ Acetylcholine esterase inhibitors (AChEI) stimulating the cholinergic system are the first line therapy for Alzheimer's disease. Studies have shown small but persisting cognitive benefits with AChEIs^20^ and reduced mortality in Alzheimer's disease.^21^ On the other hand, anticholinergic drugs have been associated with an increased risk of mortality and stroke in dementia patients.^18^ Whether this applies to inhaled anticholinergics has not been investigated.

Given the scarce research on patients suffering from both dementia and COPD, our primary objective is to characterize COPD patients—using inhaled anticholinergic use as a proxy—within the Swedish dementia registry (SveDem) and to examine differences in mortality between users and non-users. Objective measures of COPD severity are not available in Swedish register data, and the Swedish Airway Register had insufficient coverage during the study period.^22^ Consequently, we did not use this register, which limits our ability to determine whether observed mortality differences reflect pharmacological effects of inhaled anticholinergics or underlying disease severity. Nevertheless, this is the first study to investigate inhaled anticholinergics in dementia patients and to demonstrate important mortality differences, highlighting the need for further studies to clarify the mechanisms behind these findings.

## Methods

### Data sources and study design

We conducted a retrospective cohort study based on data from the Swedish registry for dementia/cognitive disorders (SveDem) and the Swedish Prescribed Drug Register, where we included data of patients in SveDem with a dementia diagnosis date between January 1, 2008 and December 31, 2017. Among these patients, we identified individuals with concomitant purchase of LAMA/SAMA, using data from the Swedish Prescribed Drug Register from January 1, 2006 until December 31, 2017. Follow-up data for all included patients were available only until December 31, 2017, which marks the end of the observation period for this study. The date of the dementia diagnosis was used as the index date.

SveDem^23^ is the world's largest quality database for patients with different dementia disorders. The registry was started on May 1, 2007. The aim of SveDem is to improve dementia treatment and care with annual follow-up. Based on estimated dementia incidence of the population irrespective of whether a dementia diagnosis is obtained or not, the coverage of SveDem for new dementia cases is estimated to 28–43%.^24^ Patient specific information regarding the type of dementia, diagnosis date, age, sex, number of drugs used, cognitive assessment with Mini-Mental State Examination (MMSE) score, Charlson Comorbidity Index (CCI), whether the patient has received the dementia diagnosis in primary or specialist care, and date of death were retrieved from SveDem. SveDem is permitted to use MMSE and has done so since 2007, so no specific license was obtained. In the calculation of CCI, the dementia diagnosis was removed since all included patients have dementia.^25,26^ Information on dispensed drugs and the time of medication purchase was retrieved from the Swedish Prescribed Drug Register^27^ held by the Swedish National Board of Health and Welfare. The register includes prescriptions dispensed in Swedish pharmacies since July 1, 2005, and has a coverage of >99%. However, drugs administered during hospital stays are not captured. In the present study information about dispensed drugs was provided from the start of the register until the last day of December 2017.

Data on underlying and contributing causes of death were retrieved from the Swedish Cause of Death Register, which covers all deaths in Sweden.

Data from the registers were linked and pseudonymized by the statistics unit at the Swedish National Board of Health and Welfare.

### Study population

Our study defines exposed and unexposed groups based on drug exposure to inhaled anticholinergics (LAMA/SAMA) rather than a clinical diagnosis of COPD. Since COPD diagnoses in Sweden are primarily recorded in primary care, which is poorly covered in available registers, we chose not to rely on diagnosis codes. Our focus is on the prevalence of inhaled anticholinergic use in a dementia cohort and its association with mortality.

Patients in the exposed group (LAMA/SAMA) were required to have been dispensed drugs with ATC codes R03AL (adrenergics with anticholinergics, including triple combinations with corticosteroids) and/or R03BB (anticholinergics) at least once per year in the two years before the index date or more than once in the year before the index date. In the primary intention-to-treat analysis, exposure classification was not updated after index; subsequent discontinuation, switching, or initiation did not alter group assignment.

The unexposed group included all other patients in SveDem with a dementia diagnosis within the study period who did not meet the criteria for the exposed group.

### Statistical methods

For descriptive statistics prevalence was reported, for continuous variables medians and IQRs were reported, and total numbers and percentages were reported for categorical variables. No patients were excluded for descriptive statistics.

**Figure 1.**
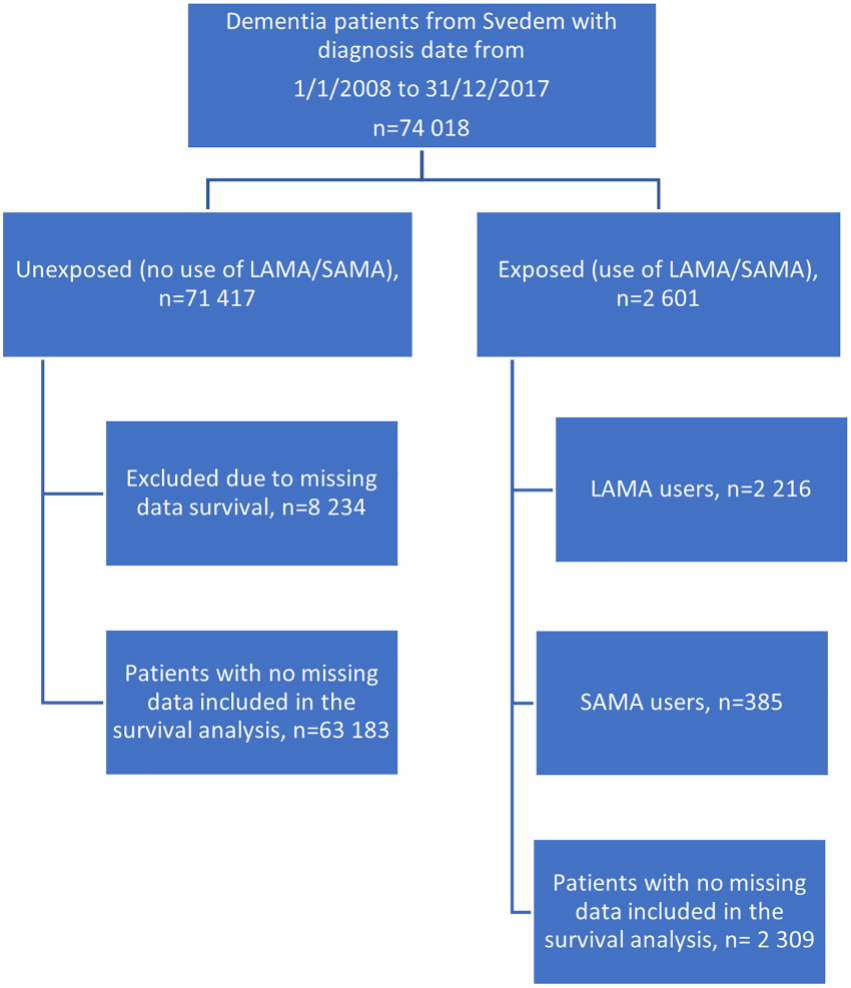
Flowchart demonstrating included patients in the two groups. The comparison group (unexposed) included 71 417 patients whereas the exposed group included 2 601 patients. Patients in the comparison group were censored in the survival analysis after first purchase of LAMA/SAMA. Patients with missing data for the covariates included in the survival analysis were excluded from the analysis. LAMA: long-acting muscarinic antagonists; SAMA: short-acting muscarinic antagonists; MMSE: Mini-Mental State Examination.

The survival distribution was assessed among dementia patients with or without LAMA/SAMA by a survival curve using the Kaplan–Meier method. Patients in the unexposed group who purchased LAMA/SAMA after the index date were censored at the date of purchase in the survival analysis. Statistical testing of differences in survival between groups was performed by Log-rank test. Cox-proportional hazard regression was used to estimate the hazard ratios, both crude and adjusted for common significant covariates identified in earlier SveDem studies.^20^^28–30^ The covariates include age, sex, type of dementia (Alzheimer's dementia, mixed dementia, vascular dementia, Lewy body dementia, frontotemporal Dementia and Parkinson's Disease Dementia), grade of dementia (mild, moderate and severe), comorbidity (CCI), type of diagnosis unit (primary or specialist care) and co-resident status). Patients with missing data for these covariates were excluded from survival analysis (Figure 1). Patients were followed for three years after the index date. Tests of proportionality of hazards were performed.

To ensure sample representativity stratification was performed based on the MMSE score at the time of the dementia diagnosis (0–10, 11–20, and 21–30), type of dementia disorder, CCI, age and sex. Each stratum was analyzed separately using Cox-proportional hazard regression. Analysis of interaction effects was also performed. In a subgroup analysis, survival was compared between the unexposed group and exposed patients stratified into LAMA and SAMA users. Patients with any LAMA purchases were classified as LAMA users, even if they also had SAMA purchases. Patients with SAMA purchase and no LAMA purchase was classified as SAMA users.

A sensitivity analysis was conducted to assess the impact of different purchase rates by comparing groups based on the frequency of LAMA/SAMA purchases. In this analysis, patients who had made three or more purchases per year in the last year before dementia diagnosis were classified as exposed. A Wald test was performed to analyse the difference between groups.

The time at risk was calculated individually for all patients in person years from dementia diagnosis to the date of death or the end of follow up, with a maximum of three years. Standardized mortality rates (SMR) were calculated by calculating the observed deaths based on person years. The observed mortality rate was then compared with the expected mortality in each age group by using data from the Swedish Central Statistics Agency (SCB) from 2015.^31^

We additionally identified and summarized the five most common underlying causes of death, both by ICD-10 groups and by specific ICD-10 codes.

### Human ethics and consent to participate declarations

Ethical approval for this study was obtained by the Swedish Ethical Review Authority (Etikprövningsmyndigheten) Dnr 2015/743-31/4, 2017/942-32, 2018/2087-32, 2024-00406-02. Patients are informed about registration in SveDem at the time of their dementia diagnosis. Patients can refuse registration, obtain information on their registration at any time or withdraw their consent. All research projects using SveDem data must be approved by the ethics committee. Signed consent for research, however, was not required for this study in accordance with the protocol submitted and approved by the ethics committee. Data were pseudonymized by Swedish authorities before delivery to the research team. Clinical trial number: not applicable.

## Results

### Demographics and prescription patterns of LAMA/SAMA in exposed and unexposed groups

In total 74 018 (59% female) patients received their dementia diagnosis between 2008-01-01 and 2017-12-31 and were registered in SveDem (Figure 1). Of them, 2 601 (3.5%) patients fulfilled the inclusion criteria for the LAMA/SAMA group (exposed group). The comparison group comprised the remainder of the cohort, 71 417 patients, who did not meet the inclusion criteria for the LAMA/SAMA group (unexposed group). The MMSE score at the index date was significantly (p = 0.007) higher in the exposed group (21.1 ± 3.5) compared to the unexposed group (20.8 ± 3.5). The demographic data are presented in Table 1.

**Table 1.** Baseline characteristics of the study population.

All variables are shown in n, (%) unless otherwise noted	Total, n = 74,018	Exposed group (LAMA/SAMA), n = 2601	Unexposed group, (nonuser) n = 71,417
Male	30 591 (41%)	1122 (43%)	29 469 (41%)
Age, years (median, IQR)	81 (75–85)	80 (75–85)	81 (75–85)
Number of drugs, median (IQR)	4 (2–7)	7 (5–10)	4 (2–7)
AChEI	39 379 (53%)	1078 (41%)	38 301 (54%)
Memantine	21 382 (29%)	666 (26%)	20 716(29%)
Type of dementia			
AD (late and early onset)	22 841 (31%)	553 (21%)	22 288 (31%)
Mixed dementia	13 778 (19%)	566 (22%)	13 212 (18%)
Vascular dementia	14 152 (19%)	653 (25%)	13 499 (19%)
LBD	1 560 (2%)	49 (2%)	1511 (2%)
FTD	1 129 (1.5%)	39 (1.5%)	1 090 (1.5%)
PDD	1 102 (1.5%)	18 (0.69%)	1 084 (1.5%)
Unspecified dementia	17 498 (24%)	654 (25%)	16 844 (24%)
Other dementia	1 958 (2.6%)	69 (2.7%)	1 889 (2.6%)
Charlson comorbidity Index, median (IQR)	2 (1–3)	3 (2–4)	2 (1–3)
MMSE-score, median (IQR)	21 (18–25)	22 (18–25)	21 (18–24)
Dementia Severity at the time of diagnosis			
Mild dementia (MMSE 20–30)	39 846 (54%)	1492 (57%)	38 354 (54%)
Moderate dementia (MMSE 10–19)	26 562 (36%)	897 (35%)	25 665 (36%)
Severe dementia (MMSE <10)	2479 (3%)	66 (3%)	2413 (3%)
Missing	5131 (7%)	146 (6%)	4 985 (7%)
Deaths within 3 years after index date	19748 (27%)	1009 (39%)	18 739 (26%)

Patients with at least one purchase of LAMA/SAMA per year the two years before index date or with more than one purchase the year prior index date were considered as LAMA/SAMA patients. The comparison group included all other patients in SveDem. LAMA: long-acting muscarinic antagonists; SAMA: short-acting muscarinic antagonists; AChEI: acetylcholinesterase inhibitors; AD: Alzheimer's disease; LBD: Lewy body dementia; FTD: frontotemporal dementia; PDD: Parkinson's disease dementia: MMSE: Mini-Mental State Examination.

Nearly 20% (n = 325) of the survived patients in the exposed group did not receive any LAMA/SAMA within three years after dementia diagnosis, while 58% (n = 924) had at least one purchase each year three years after dementia diagnosis. Within three years after the index date 1 478 (2%) of patients defined as unexposed had at least one purchase of LAMA/SAMA, these patients were censored from the survival analysis the day of purchase. Totally during the entire study period (10 years), 2 175 (3%) patients had at least one purchase of LAMA/SAMA after the index date.

### Association between LAMA/SAMA (exposed) and survival

The age adjusted mortality comparisons between the total cohort exposed, unexposed and the general Swedish population are shown in Table 2. The SMR was higher across all age groups in exposed patients compared to unexposed. The total follow-up time in person-years was 193 892 years at risk with 19 748 observed deaths (mortality rate 100 deaths/1000 person years). For the exposed group the total follow-up time in person years was 6 223 years with 1 009 observed deaths (mortality rate 162 deaths/1 000 person-years). SMR in the age category 61–75 was 8.21 (95% CI: 7.05–9.56) in the exposed group compared to 4.08 (95% CI: 3.92–4.24) in the unexposed group.

**Table 2.** Standardized mortality rates in exposed, unexposed, and the whole cohort compared to the general Swedish population (expected deaths) based on different age groups.

	ALL				Exposed				Unexposed			
**Age**	Observed deaths	Expected	SMR	CI 95%	Observed deaths	Expected	SMR	CI 95%	Observed deaths	Expected	SMR	CI 95%
0–60	0.022	0.001	21.99	18.1–26.7	0.068	0.001	68.1	25.56–181	0.022	0.001	21.384	17.48–26.14
61–75	0.051	0.012	4.21	4.05–4.38	0.098	0.012	8.21	7.05–9.56	0.049	0.012	4.080	3.92–4.24
75–90	0.113	0.06	1.88	1.85–1.92	0.177	0.06	2.94	2.75–3.16	0.111	0.060	1.845	1.81–1.88
90+	0.265	0.241	1.1	1.06–1.14	0.405	0.241	1.68	1.34–2.11	0.262	0.241	1.086	1.04–1.13

Population data was obtained from the Statistics Sweden (SCB) for the year 2015.

**Figure 2.**
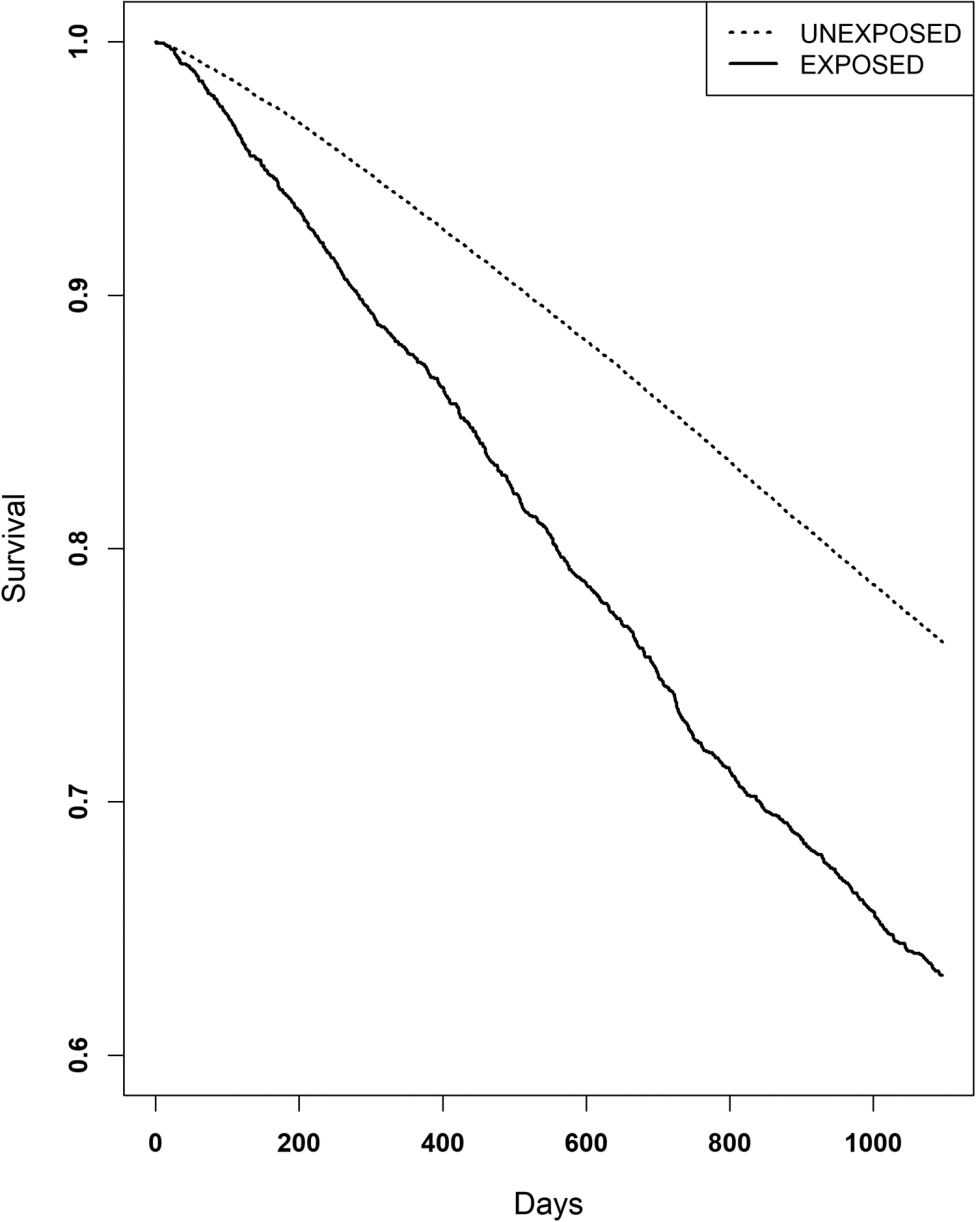
Kaplan-Meier curve illustrating survival. Patients with dementia and purchased LAMA/SAMA had a greater mortality (unadjusted HR 1.73, 95% CI: 1.62–1.86, p < 0.0001).

All patients with complete data were included in the survival analysis, n = 65 492. Survival curves are illustrated in Figure 2, which shows the overall survival per group. Log-rank test showed a significant difference in survival between the groups (p < 0.0001), with an unadjusted hazard ratio (HR) of 1.73 (95% CI: 1.62–1.86, p < 0.0001). Cox-regression showed a significantly greater risk of death in the exposed group compared to the unexposed group (HR 1.39, 95% CI: 1.29–1.49, p < 0.0001) adjusted for age, sex, MMSE-score at baseline (grade of dementia), type of dementia, type of diagnostic unit (primary or specialist care), coresident status and comorbidity (CCI) (Table 3).

**Table 3.** Cox regression survival, the unexposed group was used as the reference group.

Survival	Unadjusted, N = 65 492	Adjusted, N = 65 492
**HR (95% CI)**	1.73 (1.62–1.86) ***	1.38 (1.29–1.48) ***
**Deaths within 3-years after the index date, n (%)**	15 653 (24%)	15 653 (24%)

Adjusted and unadjusted hazard ratios (HR) with 95% confidence intervals (CI) and p-value are shown. The analysis was adjusted for age, sex, type of dementia, Mini-Mental State Examination (MMSE) at dementia diagnosis date, type of residency, co-residency and comorbidity measured by Charlson comorbidity index (CCI). ***p < 0.0001.

**Figure 3.**
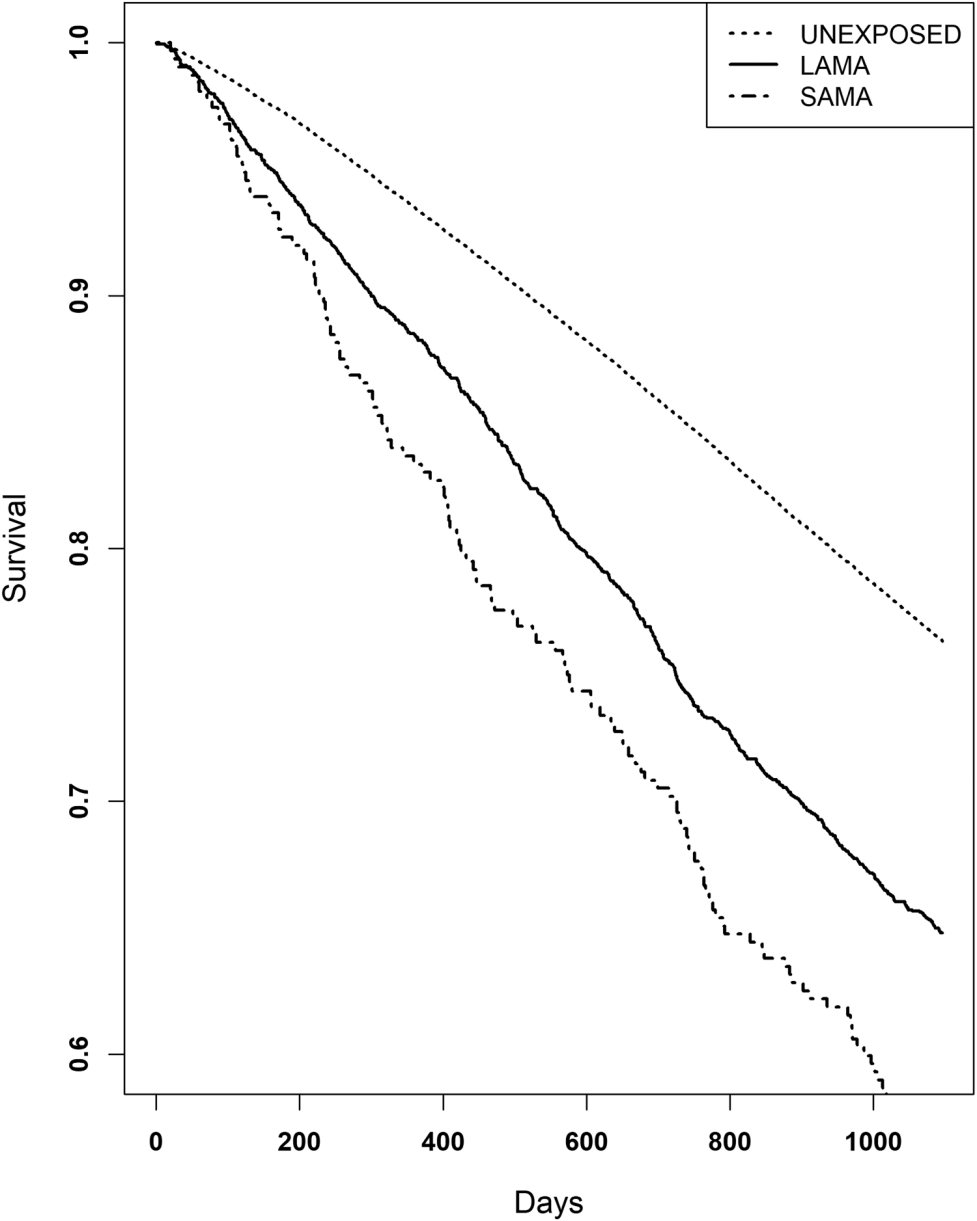
Kaplan-Meier for long-acting inhaled anticholinergics (LAMA) and short-acting inhaled anticholinergics (SAMA) analyzed separately. Patients with dementia and exposed to SAMA had an increased mortality risk, Hazard ratio (HR) 2.26, (95% CI: 1.87–2.72, p < 0.0001) compared to unexposed. Dementia patients exposed to LAMA also had an increased mortality risk compared to unexposed (HR 1.68, 95% CI: 1.56–1.80, p < 0.0001), but a lower risk compared to patients exposed to SAMA.

In a subgroup analysis, the exposed group was divided into SAMA and LAMA users (Figure 3). Compared with the unexposed group, SAMA users had a higher mortality risk (HR 2.19, 95% CI: 1.83–2.61, p < 0.0001) than LAMA users (HR 1.64, 95% CI: 1.52–1.76). After adjustment for covariates, the risk remained elevated for both groups, with an adjusted HR of 1.64 (95% CI: 1.34–2.01, p < 0.0001) for SAMA users (Table 4).

**Table 4.** Cox regression for subanalysis of survival, the unexposed group was used as the reference group.

Survival, subanalysis exposed group	SAMA, N = 246	LAMA, N = 2 061
HR (95% CI)		
Adjusted	1.79 (1.48–2.16)***	1.34 (1.24–1.44)***
Unadjusted	2.26 (1.87–2.72)***	1.68 (1.56–1.80)***
Deaths within 3-years after the index date, n (%)	110 (44%)	741 (29%)

Adjusted and unadjusted hazard ratios (HR) with 95% confidence intervals (CI) and p-values are shown. The analysis was adjusted for age, sex, type of dementia, Mini-Mental State Examination (MMSE) at basal examination, type of diagnosis unit (primary or specialist care), co-residency and comorbidity measured by Charlson comorbidity index (CCI). ***p < 0.0001.

A sensitivity analysis based on different purchase rates showed an increased mortality in patients with more frequent purchases, defined as three or more purchases the last year prior to the index date (HR 1.83, 95% CI: 1.69–1.98, p < 0.001) compared to patients with non-continuous purchases (HR 1.49, 95% CI: 1.29–1.70, p < 0.001) (Supplemental Table 1 and Supplemental Figure 1). Using the Wald test, we found a statistically significant difference between the HRs (Wald χ² = 6.8, p = 0.009).

Table 5 shows survival after stratification upon type of dementia diagnosis, CCI, dementia severity, sex, and age. The mortality in the exposed group was significantly higher in most strata, which is in line with the calculated HR. Interaction analysis did not show any significant interaction between the covariates and different strata.

**Table 5.** Results of Cox proportional hazard regression examining mortality associated with baseline factors adjusted for age, gender, MMSE (Mini-Mental State Examination), type of dementia, type of diagnosis unit (primary or specialist care), co-resident status and co-morbidity. Unexposed patients in each category served as reference.

	HR Exposed (95% CI)	*p*	Deaths, (%)
**Gender, n**			
Female, 38 163	1.43 (1.30–1.58)	<0.0001***	8 146 (21.3)
Male, 27 329	1.33 (1.21–1.48)	<0.0001***	7 507 (27.5)
**Age, n**			
0–60, 1 290	3.13(0.91–10.71)	0.07	71 (5.5)
61–75, 16 856	1.57 (1.32–1.87)	<0.0001***	2 174 (12.9)
76–90, 44 142	1.34 (1.24–1.46)	<0.0001***	11 788 (26.7)
>91, 3 204	1.38 (1.03–1.83)	0.026*	1 620 (50.6)
**MMSE, n**			
0–10, 2 096	1.72 (1.20–2.50)	0.003*	910 (43.4)
11–20, 24 667	1.30 (1.16–1.44)	<0.0001***	7 587 (30.8)
21–30, 38 729	1.44 (1.30–1.58)	<0.0001***	7 156 (18.5)
**Type of dementia, n**			
AD, 21 266	1.60 (1.36–1.87)	<0.0001***	3 520 (16.5)
MIXD, 12 675	1.41 (1.23–1.63)	<0.0001***	3 455 (27.3)
VaD, 12 257	1.33 (1.17–1.52)	<0.0001***	3 701 (30.2)
UNSD, 14 351	1.27 (1.09–1.48)	<0.0019**	3 749 (26.1)
**CCI, n**			
I, 30 767	1.14 (0.85–1.53)	0.3871	5 392 (17.5)
II, 14 142	1.57 (1.36–1.81)	<0.0001***	3 455 (24.4)
III, 10 433	1.41 (1.21–1.64)	<0.0001***	2 930 (28.1)
>III, 10 150	1.35 (1.22–1.49)	<0.0001***	3 876 (38.2)

Results are presented as hazard ratios (HR), 95% confidence intervals (CI) for relative mortality and p-values for parameter estimates (Cox regression). The number of deaths within three years after baseline are presented in each stratum. AD: Alzheimer's disease; MIXD: Mixed dementia; VaD: Vascular dementia; UNSD: Unspecified dementia.

In the cohort overall, circulatory diseases were the leading cause of death by ICD group. When considering specific ICD codes, dementia was the most common cause of death in the total cohort and in the unexposed group, while COPD was the most frequent single cause of death in the exposed group (n = 215 (21%)).

## Discussion

We found that people living with dementia and COPD, identified by use of SAMA/LAMA, had a significantly decreased survival compared to dementia patients not using SAMA/LAMA. The standard mortality rate was also higher for dementia patients using SAMA/LAMA compared to unexposed dementia patients and the general Swedish population.

It is well-established that an increased anticholinergic burden is associated with a greater risk of dementia and other morbidities.^32,33^ Previous research on SveDem patients has demonstrated a relationship between high anticholinergic burden and increased risk of stroke and death,^18^ but inhaled anticholinergics were not included. However, all inhaled anticholinergics are systemically absorbed, either through the pulmonary system or the gastrointestinal tract. Systemic concentrations of all these substances have been measured.^34–47^ Therapeutic concentrations have been detected in blood and urine, and systemic side effects such as urinary retention and blurred vision have been reported.^48,49^ Additionally, some studies have indicated an increased risk of cardiovascular events associated with inhaled anticholinergics in COPD patients.^32^ It's known that the permeability of the blood-brain-barrier (BBB) also increases with increasing age and comorbidities.^50,51^ Older patients with dementia may be more vulnerable because of a dysfunctional and more permeable BBB,^50,52,53^ potentially facilitating central anticholinergic effects.

The lower survival observed in the exposed group is most likely mainly attributed to the indication for inhaled anticholinergics, COPD. It is well known that patients with COPD have poorer survival compared with the general population, as well as with dementia patients without COPD.^54–57^ Some studies have suggested beneficial survival effects of inhaled anticholinergics in COPD patients,^58^ while others have reported a dose-dependent increased mortality.^59^ Unfortunately, with this study design it is not possible to discern whether the lower survival is solely due to the association with COPD, or whether there may be negative effects of inhaled anticholinergics in this population. Our analysis of causes of death provides further context. Although COPD-related deaths were more frequent among exposed patients, they were not the predominant cause. Cardiovascular causes such as acute myocardial infarction and dementia itself were more frequent together. This suggests that circulatory and dementia-related comorbidities substantially contribute to mortality, and that the increased mortality observed among exposed patients cannot be explained by COPD alone, underscoring the complexity of disentangling disease burden from treatment effects.

Survival was significantly lower among SAMA users compared with LAMA users. SAMA (ipratropium alone or in combination with salbutamol) is a short acting anticholinergic drug, primarily used for symptom relief and has pharmacokinetics similar to other inhaled anticholinergics but with shorter duration of action.^48^ Prior studies have reported conflicting results regarding the effect of ipratropium on mortality. A study on COPD and asthma patients in Denmark, showed that ipratropium use was associated with a significantly higher mortality both in COPD (adjusted HR 1.6) and asthma (adjusted HR 2.4) even after adjusting for several factors such as disease severity.^60^ Other studies have also reported increased cardiovascular mortality related to ipratropium use.^61^ In our cohort, the higher mortality among SAMA users (who had no LAMA purchase within three years prior to dementia diagnosis) was still prominent after adjusting for several covariates such as comorbidity. It is plausible that, among patients with cardiovascular disease (and thus higher baseline mortality), concerns about reported cardiovascular adverse effects of β2-agonists lead to more cautious use, potentially shifting prescribing toward SAMA. Nevertheless, frequent use of short-acting bronchodilators may be a marker of more frequent symptoms of obstructive lung diseases. This has been described for use of SABA in asthma, where frequent use of SABA is associated with poor outcome^62^ as well as in COPD.^63^ From the present study, it is not possible to differentiate between a possible negative effect of SAMA on survival, or whether SAMA use is a marker of undertreatment or a COPD phenotype with more frequent need for symptomatic relief. Yet another hypothesis would be that the poor survival is entirely attributed to COPD and that use of LAMA has a beneficial effect on survival in COPD. A beneficial effect of LAMA on survival in COPD has previously been suggested.^58^

In line with what has been described in earlies studies,^64^ we could see an irregular purchase of LAMA/SAMA medications in our cohort. This is important to consider, because this patient group has poor adherence and persistence to drug therapy consequently leading to irregular drug purchases.^64,65^ Evidence of this became apparent in our study, with only 924 patients (58%) out of 1 592 survived exposed patients having at least one annual purchase of LAMA/SAMA three years post index date which is a small proportion. In contrast, only 325 patients out of the 1 592 (20%) exposed patients did not purchase LAMA/SAMA during the same three-years period after index-date, indicating a markedly irregular drug purchase pattern within this group. Given the irregular dispensing patterns observed, clinicians should be aware of potential adherence challenges in this population. Non-adherence to inhaled anticholinergics could reduce their therapeutic efficacy while increasing the risk of adverse outcomes. This highlights the need for closer monitoring and perhaps patient education on medication adherence, particularly in elderly patients with dementia who may have difficulty managing complex treatment regimens. Interestingly, poor adherence per se, even to placebo, has also been associated with poor outcomes.^66^

Finally, a certain percentage of the unexposed group purchased LAMA/SAMA after their dementia diagnosis. This could be due to increased contact with healthcare and hence pulmonary symptoms may be identified and treated. The increase could also be due to pulmonary adverse effects of common dementia drugs. AChEI may theoretically increase obstructivity as a concentration-dependent side effect. Further studies are needed to investigate the relationship between use of AChEI and initiation of inhaled anticholinergics, or even inhaled bronchodilators.

A major strength of our study is the large sample size, long follow-up and the good representativeness. We had few exclusion criteria and aimed to describe real-life dementia patients treated with inhaled anticholinergics. Analyzing patients upon dispensed drugs rather than disease occurrence is advantageous, as COPD diagnosis codes are not always documented in available registers in Sweden, making our drug-based analysis more comprehensive. Furthermore, according to Swedish clinical guidelines during the study period, LAMA/SAMA treatment was primarily recommended for COPD patients. Tiotropium could be considered as add-on therapy for severe asthma from 2015, but such cases are expected to be rare. Therefore, we believe that with our definition of exposure, we mainly captured patients with COPD.^7,67^

However, our study also has several limitations. In this dataset, it was not possible to identify a control group of COPD patients without inhaled anticholinergics. Had it been possible, it might still not have provided a relevant comparison due to the specific characteristics likely influencing the decision to avoid anticholinergic treatment. In future studies, incorporating disease severity data, such as lung function measures, clinical severity indices, and patient reported outcomes, could help address this confounding by allowing for more precise adjustment for patient health status, thus improving the validity of the observed associations. Additionally, there may be residual confounding due to unmeasured or imprecisely measured confounders that could influence the observed associations such as smoking and other life-style factors. Since smoking is strongly associated both with COPD incidence and with mortality, the absence of this information is a major limitation. Other lifestyle factors such as body mass index, frailty, and socioeconomic status may further bias our results. Consequently, we cannot exclude that part of the observed association reflects these unmeasured factors.

The irregular purchase pattern complicated accurate classification of exposure status and posed a methodological challenge. Because we used an intention-to-treat design, exposure was defined only at baseline and not updated thereafter. This approach introduces potential misclassification: patients with irregular pre-diagnosis purchases may have been classified as unexposed. Since we did not follow purchase behavior after the index date, we cannot be certain of actual exposure status during follow-up. Possible discontinuation after dementia diagnosis is not regarded as a major methodological problem in our study, since exposure is used as a proxy for the chronic disease COPD. However, it could affect results that were due to effects of the medication.

Our analysis is based on registry data on dispensed prescriptions. Patients who failed to redeem their prescriptions thus will have been classified as unexposed and thus wrongly assumed not to have COPD. Finally, while our study benefits from a large sample size, the SveDem data had full coverage of specialist care but less coverage of primary care. Thus, our findings may not be representative of dementia patients diagnosed only in primary care.

In conclusion, inhaled anticholinergic use in people living with dementia was associated with higher mortality compared with non-use. The association may reflect COPD severity, residual confounding (e.g., smoking), or possible medication effects. Further research with detailed clinical data on COPD severity, smoking, and adherence is needed to clarify these relationships.

## Supplemental Material

sj-docx-1-alz-10.1177_13872877251406309 - Supplemental material for Increased mortality in dementia patients using inhaled anticholinergics: A nationwide register study from the Swedish registry on dementia/cognitive disorders, SveDem Supplemental material, sj-docx-1-alz-10.1177_13872877251406309 for Increased mortality in dementia patients using inhaled anticholinergics: A nationwide register study from the Swedish registry on dementia/cognitive disorders, SveDem by Suzan Al-Mayahi, Marine L Andersson, Minjia Mo, Sara Garcia-Ptacek, Hong Xu, Eva Wikström and Maria Eriksdotter in Journal of Alzheimer's Disease
