# Supplementary material for: Increased mortality in dementia patients using inhaled anticholinergics: A nationwide register study from the Swedish registry on dementia/cognitive disorders, SveDem
Source: J Alzheimers Dis. 2025 Dec 16;109(3):1306–18. doi: 10.1177/13872877251406309 (PMC12855612; doi:10.1177/13872877251406309)
Supplement: sj-docx-1-alz-10.1177_13872877251406309 - Supplemental material for Increased mortality in dementia patients using inhaled anticholinergics: A nationwide register study from the Swedish registry on dementia/cognitive disorders, SveDem [file sj-docx-1-alz-10.1177_13872877251406309.docx]

**Supplemental Material**

**Increased mortality in dementia patients using inhaled anticholinergics: A nationwide register study from the Swedish registry on dementia/cognitive disorders, SveDem**

**Supplemental Table 1**. Unadjusted and adjusted hazard ratios from Cox regression based on different purchase rates.

| **Survival, sensitivity analysis based on purchase rate** | **Continuous exposure**  **N=1 903** | **Not cont­inuous exposure**  **N=698** |
| --- | --- | --- |
| **HR (95% CI)** |  |  |
| **Unadjusted** | 1.83 (1.69-1.98)*** | 1.49 (1.29-1.70)*** |
| **Adjusted** | 1.46 (1.34-1.58)*** | 1.19 (1.03-1.36)* |

Hazard ratios (HR) for Patients with Continuous Exposure to LAMA/SAMA Compared to Patients with Non-Continuous Exposure to LAMA/SAMA. Continuous exposure is defined as at least three purchases in the last year before diagnosis. Non-continuous exposure refers to patients with fewer than three purchases the last year prior diagnosis.

**Supplemental Figure 1.** Kaplan-Meyer curve illustrating survival.


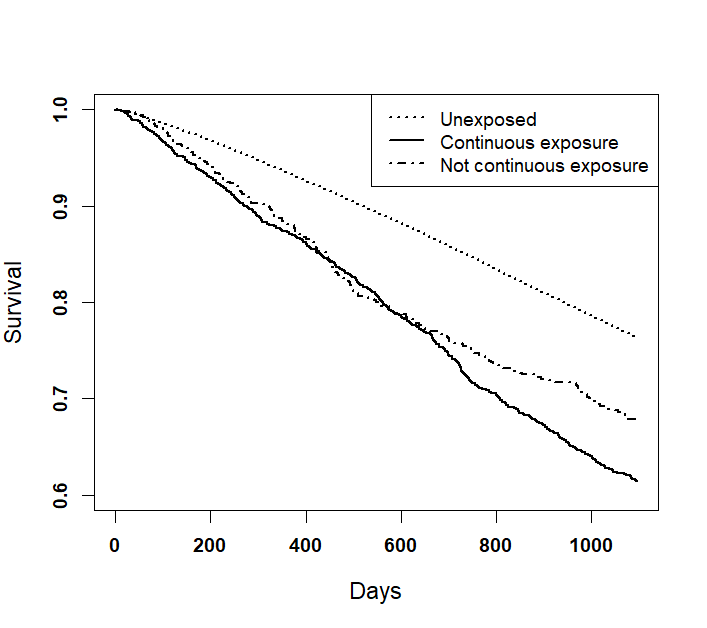


Patients with dementia who purchased LAMA/SAMA continuously had a higher mortality (unadjusted HR 1.83, 95% CI: 1.69–1.98, p<0.001) compared to patients with non-continuous purchases (unadjusted HR 1.49, 95% CI: 1.29–1.70, p<0.001) where the unexposed group served as reference.
